# Spontaneous Internal Carotid Dissection Presenting With Pulsatile Tinnitus and Hearing Loss

**DOI:** 10.7759/cureus.89099

**Published:** 2025-07-30

**Authors:** Binisha Joshi, Paul Bolaji, Yae Chun, Ahmet Ubur, Krishna Prasad Lakshminarayana Hoskere

**Affiliations:** 1 Internal Medicine, Stroke Medicine, Dorset County Hospital, Dorchester, GBR; 2 Neurology, Stroke Medicine, Dorset County Hospital, Dorchester, GBR; 3 Stroke Medicine, Dorset County Hospital, Dorchester, GBR

**Keywords:** atypical stroke presentation, cranial nerve palsy, facial nerve palsy, horner’s syndrome, internal carotid artery dissection, magnetic resonance angiography (mra), pulsatile tinnitus, trigeminal nerve

## Abstract

Internal carotid artery (ICA) dissection often presents with headache and neck pain, along with symptoms related to the anterior circulation if stroke occurs. Less commonly, it may cause Horner's syndrome and involvement of the lower cranial nerves (IX, X, XI, and XII). However, it is rare for ICA dissection to present with pulsatile tinnitus and hearing loss, which is typically linked to vertebral artery dissection. We report a rare case of a man in his 50s who initially presented to the Otolaryngology Department with a brief episode of high-pitched pulsatile tinnitus and hearing loss, preceded by left-sided headache, ptosis, dysarthria, and dysphagia. Neurological examination revealed facial numbness in the trigeminal nerve territory, mild left facial weakness, and left sensorineural hearing impairment. Magnetic resonance angiography (MRA) confirmed a left ICA dissection without evidence of vertebral artery involvement. He was managed conservatively with dual antiplatelet therapy and scheduled for follow-up computed tomography (CT) angiography. To the best of our knowledge, this is the first case report highlighting the simultaneous involvement of cranial nerves V, VII, VIII, IX, and X, along with Horner’s syndrome, due to ICA dissection.

## Introduction

Cervical artery dissection usually affects either the anterior circulation through the internal carotid artery (ICA) or the posterior circulation via the vertebral artery. It results from an arterial intimal tear, which may occur spontaneously or following trauma, leading to the formation of a false lumen, pseudoaneurysm, thrombosis, embolism, or, in rare cases, intracranial haemorrhage, which can cause neurological deficits [1].

Its incidence is 2.5-3 per 100,000 and accounts for 2% of ischaemic strokes, primarily affecting young to middle-aged adults [2]. It is one of the most important causes of stroke in individuals under 50 years of age, accounting for up to 25% of ischaemic strokes in this age group [3].

Presentations range from asymptomatic to ipsilateral headache, facial or ocular pain, neck pain, and strokes (~67%), with Horner’s syndrome seen in up to 58% [4]. Rarely, it may present with tinnitus and multiple cranial neuropathies. Headache is the most frequent early symptom and is often characterised by retro-orbital or frontotemporal distribution. In ICA dissection, it can also be associated with facial pain or partial Horner's syndrome without anhidrosis, due to disruption of postganglionic sympathetic fibres [5,6].

Timely diagnosis can be challenging due to the wide range of symptoms that often resemble less serious conditions such as migraines or Bell’s palsy. While most ear-related issues cause non-pulsatile tinnitus, pulsatile tinnitus is an important but frequently missed sign of vascular problems. It is commonly associated with venous sinus stenosis or dural arteriovenous fistulas, but ICA dissection can also present with pulsatile tinnitus, reported in up to 27% of cases in some studies [7,8].

This case demonstrates a rare spontaneous ICA dissection, where the patient’s main initial symptoms were pulsatile tinnitus and sensorineural hearing loss, features more often linked to vertebrobasilar issues. The vascular origin was only confirmed after mild neurological signs, which prompted additional neuroimaging, including MRI with fat-suppressed T1-weighted sequences and MRA, which remains the definitive diagnostic method for confirmation of the diagnosis [9,10]. Early detection and treatment are crucial to avoid complications such as cerebral infarction, which can occur in 50-80% of cases if not promptly managed [2,9].

## Case presentation

A man in his 50s presented to the Otolaryngology Department with left-sided pulsatile tinnitus and hearing loss, preceded by a week-long, left-sided headache and deep burning facial pain. The headache, described as "pressure-like," was accompanied by ptosis, dysarthria, dysphagia, and left facial droop. He had no recent trauma, chiropractic manipulation, or vertigo. His past medical history included hypertension and psoriasis; he was a non-smoker. His general practitioner (GP) initially prescribed antibiotics and steroids for presumed Bell’s palsy.

**Figure 1:**
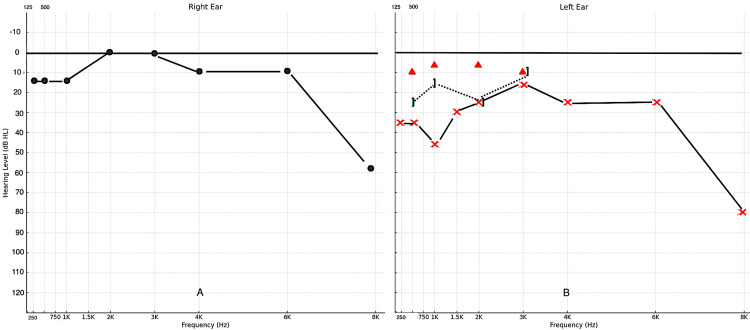
Pure-tone audiometry results (A) Right ear showing predominantly normal hearing, except for mild high-frequency loss. (B) Left ear mild sensorineural hearing loss ⚫️ (black circle): Air conduction in right ear (unmasked), ❌ (red cross): Air conduction in left ear (unmasked), 🔺(red triangle): Bone conduction in the left ear (unmasked), ] (black right square bracket): Bone conduction in the left ear (masked)

**Figure 2:**
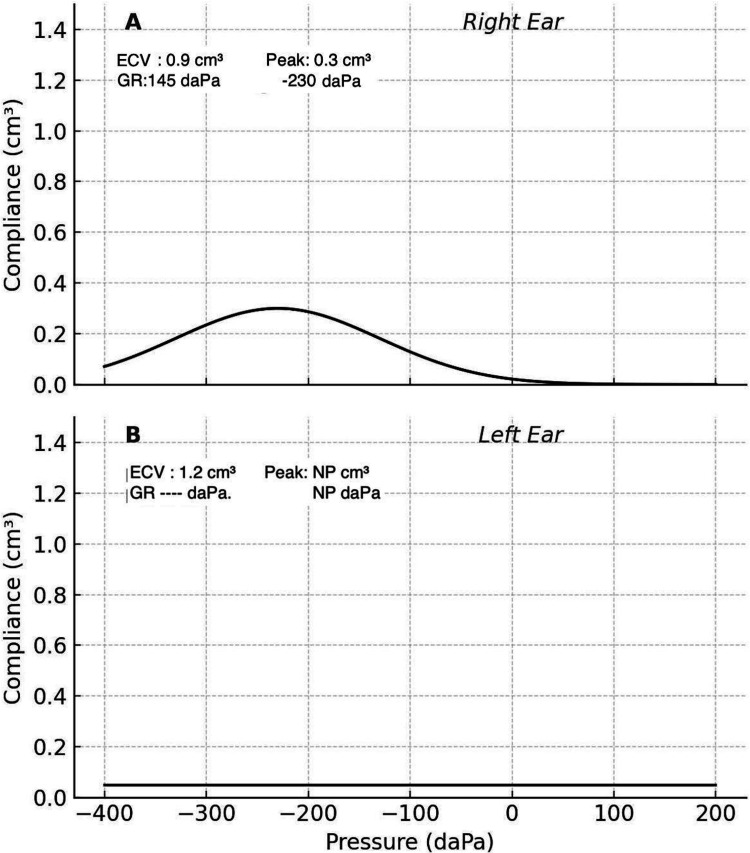
Tympanometry results (A) Right ear: Type C tympanogram indicating negative middle ear pressure or Eustachian tube dysfunction. (B) Left ear: Type B tympanogram indicating fluid in the middle ear, tympanic membrane perforation or wax. ECV: Ear canal volume, NP: Not present, GR: Gradient, daPa: decapascals

Ear, nose, throat (ENT) examination revealed reduced trigeminal sensation (V1-V3), mild left facial weakness (House-Brackmann grade 2), and anisocoria with a miotic but reactive left pupil. Initial investigation with flexible nasal endoscopy revealed no pathologies to the upper aerodigestive tract. His audiometry showed predominantly normal hearing on the right, except for high frequencies, and a mild loss on the left, which was predominantly sensorineural (Figure 1). Simultaneously, tympanometry was performed, revealing a type C curve on the right and a type B curve on the left (Figure 2).

**Figure 3:**
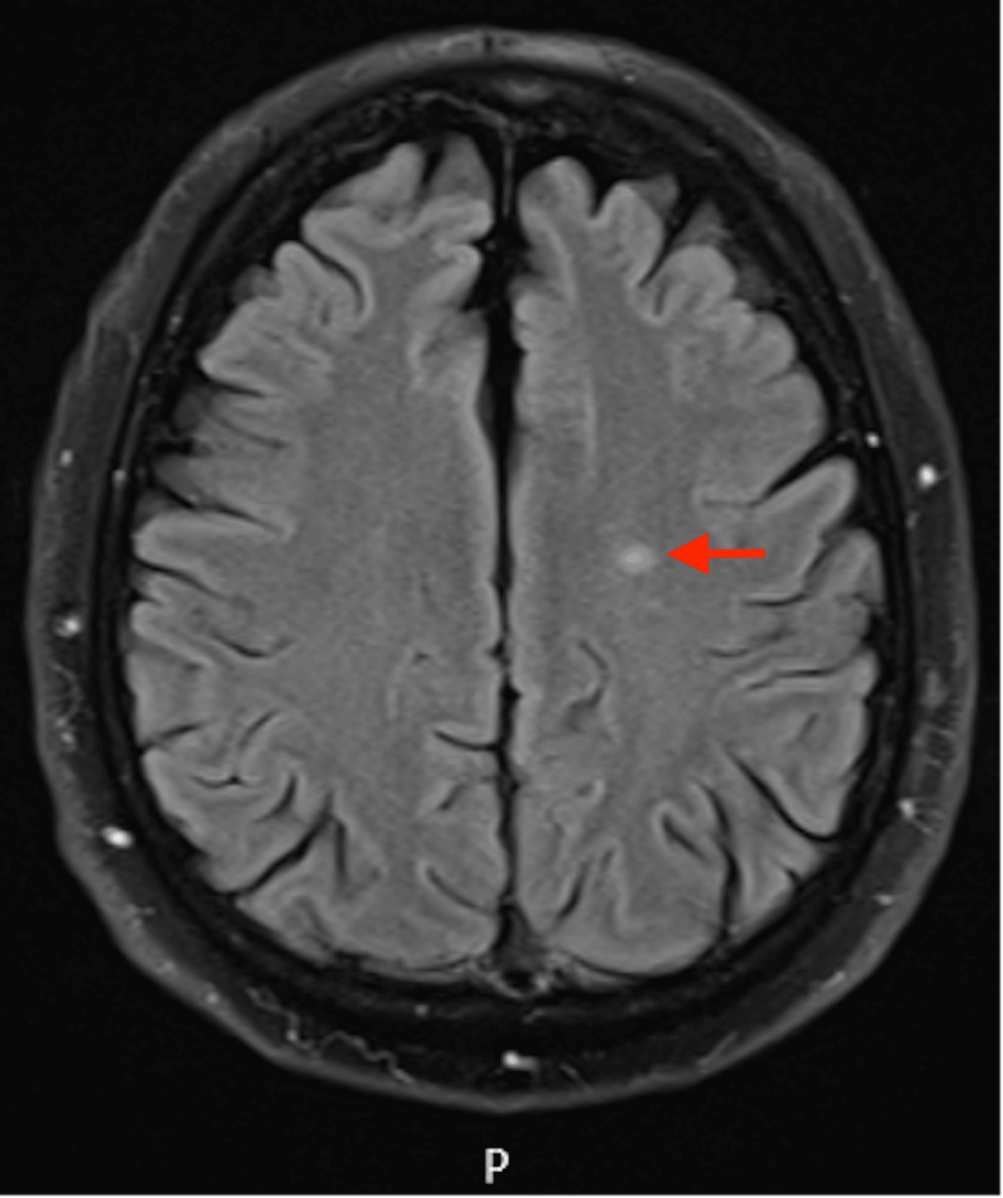
Axial FLAIR MRI showing a small infarct in the left centrum semiovale FLAIR: Fluid-attenuated inversion recovery

**Figure 4:**
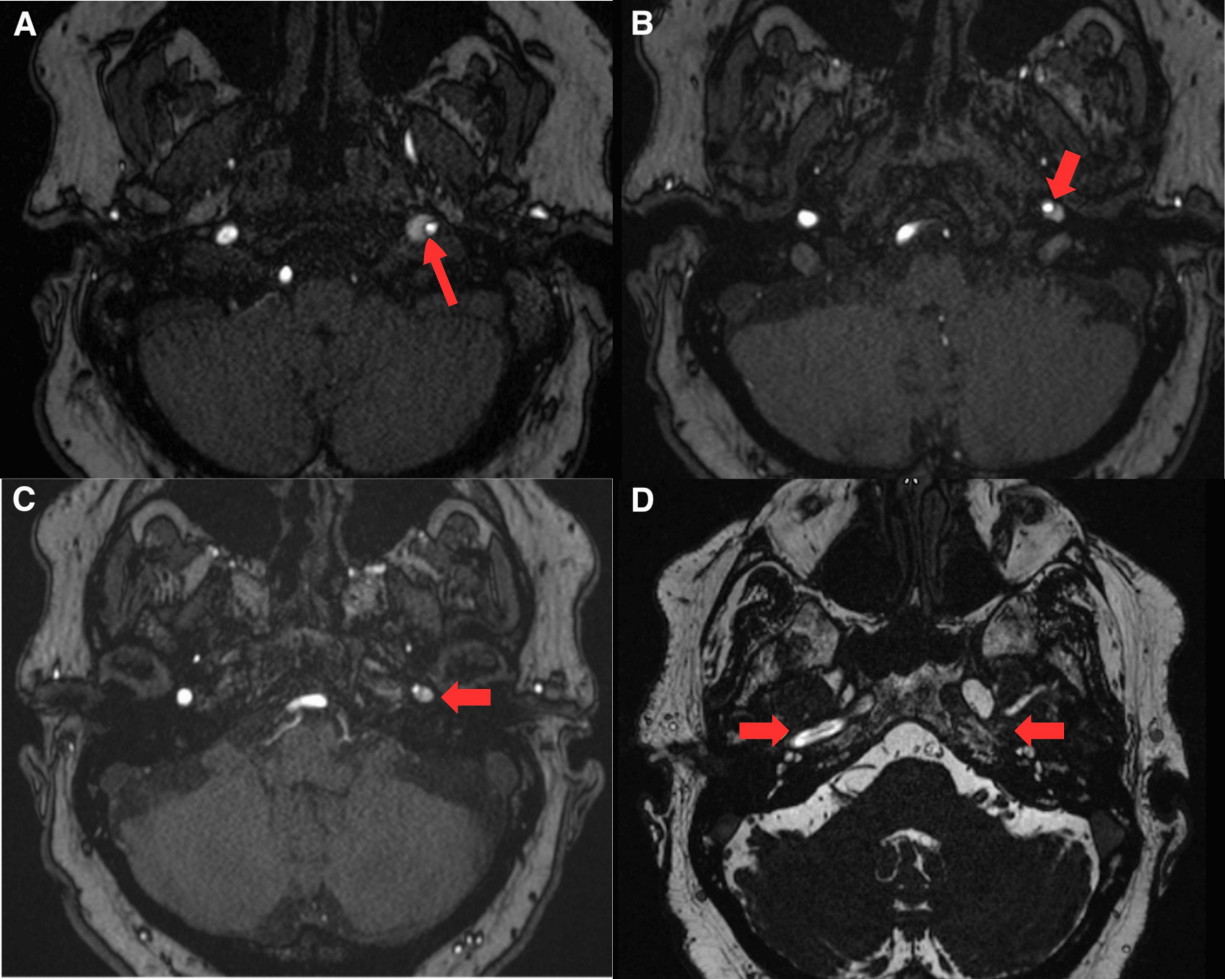
Time-of-flight (TOF) MRI angiography showing dissection of the left ICA in multiple cross-sections from the neck to the skull base (A-C) Demonstrates left internal carotid artery (ICA) in multiple cross-sections at the neck level, and (D) shows the skull base level with a patent right ICA and a stenosed left ICA secondary to dissection.

**Figure 5:**
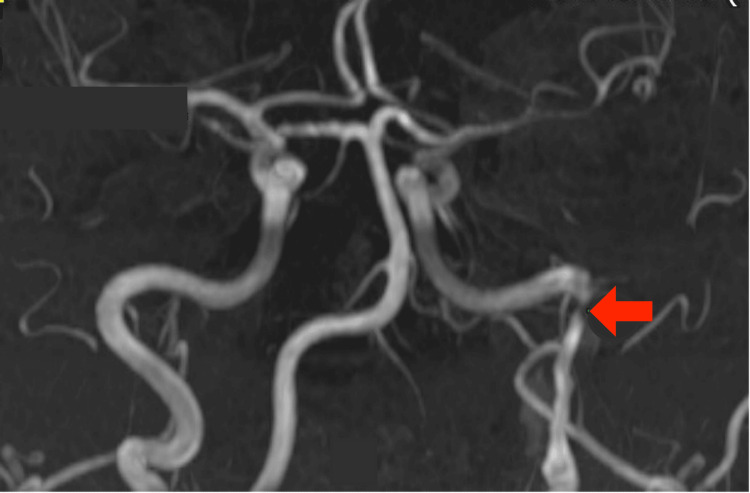
Reconstructed MRI angiography (bone window excluded) demonstrating a long-segment dissection with associated stenosis of the left ICA, extending from the cervical region to the skull base

Further imaging was requested to rule out a possible central neurologic cause given his atypical symptoms. The MRI brain scan revealed a left centrum semiovale minor infarction (Figure 3). Head and neck MRA showed a dissection of the left ICA extending from the neck to the skull base (Figure 4). There is also a figure representing the reconstructed MRI angiography (bone window excluded), demonstrating a long-segment dissection with associated stenosis of the left ICA, extending from the cervical region to the skull base (Figure 5).

His ECG showed a normal sinus rhythm. Routine blood tests were mainly unremarkable, but his lipid profile revealed elevated low-density lipoprotein (LDL) cholesterol and non-high-density lipoprotein (HDL) cholesterol levels (Table 1).

**Table 1:** Assessment of cholesterol parameters before treatment: LDL-cholesterol and non-HDL cholesterol LDL: Low-density lipoprotein, HDL: High-density lipoprotein

Variables	Results	Reference Range
LDL Cholesterol	3.4 mmol/L	< 3.0 mmol/L
Non-HDL Cholesterol	5.3 mmol/L	< 4.0 mmol/L

Elevated lipid levels can augment the risk of atherosclerosis, potentially impacting the vascular wall and increasing the likelihood of a spontaneous dissection. Although a direct causal relationship between dyslipidaemia and spontaneous dissection has not been conclusively established, the presence of modifiable cardiovascular risk factors substantiates the notion of an inherent susceptibility to arterial damage.

The patient was initially treated as Bell’s palsy based on mild facial weakness, but the MRI-detected infarct prompted further evaluation. Vertebral artery dissection and anterior inferior cerebellar artery (AICA) infarction were considered but excluded by imaging. MRA confirmed ICA dissection, correlating with the observed cranial neuropathies and pulsatile tinnitus.

**Figure 6:**
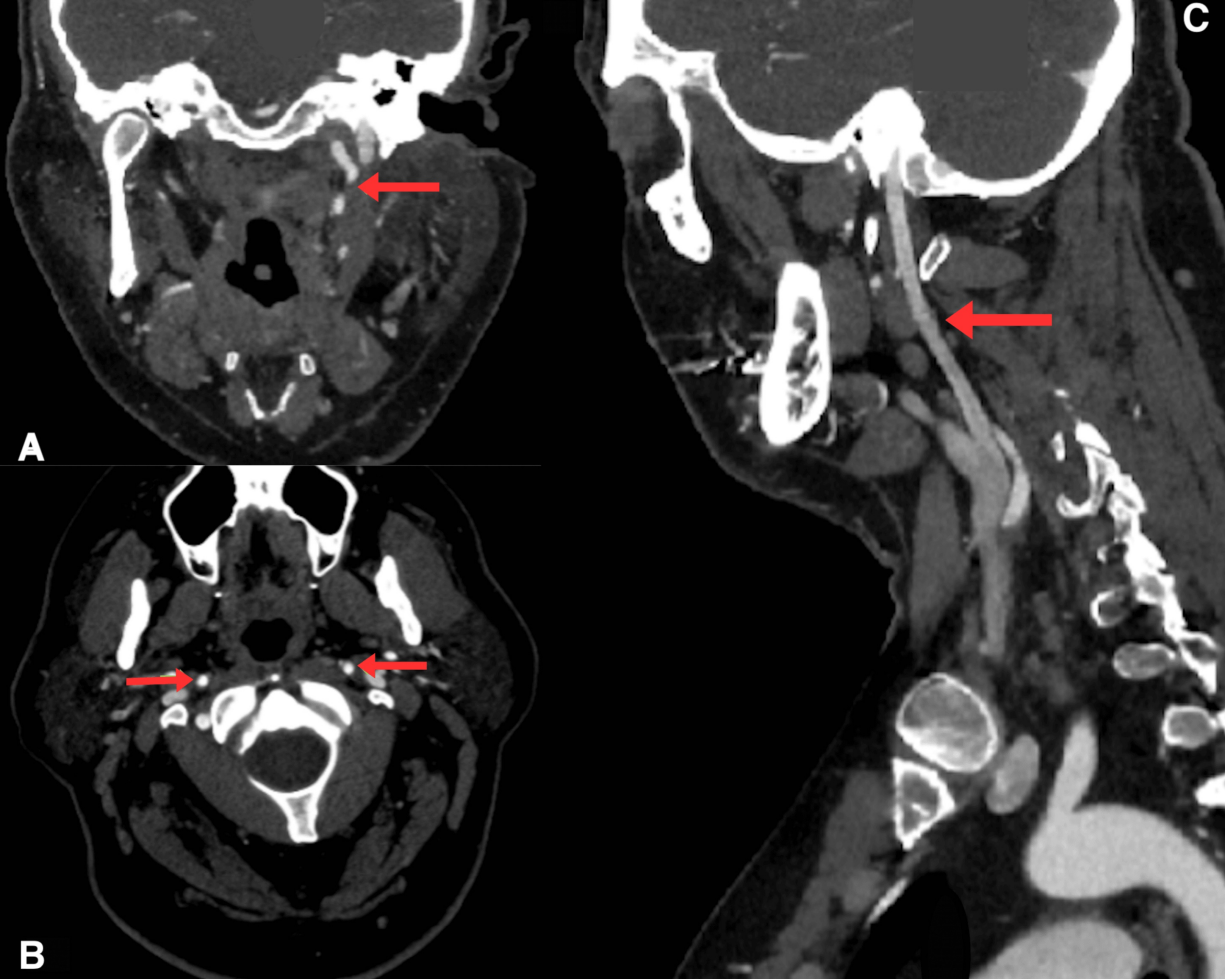
CT angiography demonstrating recanalisation of the ICA following dissection, observed at six-month follow-up (A) Coronal CT angiography showing recanalisation of the left ICA, (B) axial slice demonstrating the right ICA with recanalisation of the left ICA with symmetrical blood flow in the ICA, and (C) sagittal view showing left ICA recanalisation

The patient was then treated with dual antiplatelet therapy, aspirin 75 mg and clopidogrel 75 mg for 21 days, followed by clopidogrel alone for six months and atorvastatin 40 mg daily. A follow-up CT angiogram was scheduled for six months to assess vessel recanalisation (Figure 6), which showed improvement and recanalisation. Blood tests also indicated an improvement in his lipid profile, including LDL cholesterol and non-HDL cholesterol levels (Table 2).

**Table 2:** Assessment of cholesterol parameters after treatment: LDL cholesterol and non-HDL cholesterol LDL: Low-density lipoprotein, HDL: High-density lipoprotein

Variables	Results	Reference Range
LDL Cholesterol	2.7 mmol/L	< 3.0 mmol/L
Non-HDL Cholesterol	3.7 mmol/L	< 4.0 mmol/L

## Discussion

This case demonstrates an unusual ICA dissection presentation mimicking multiple cranial neuropathies with VIII nerve and Horner’s syndrome involvement. The patient’s left-sided headache and facial burning pain are common but nonspecific to ICA dissection. Cranial nerve palsies occur in 8-16% of cases, primarily affecting the lower nerves, while pulsatile tinnitus is reported in 16-27% of cases [9]. Sensorineural hearing loss and tinnitus shifted focus toward otological or viral causes before vascular pathology was recognized. 

Horner’s syndrome, marked by the left miotic pupil, is a frequent feature in ICA dissection [5]. The absence of neck pain or focal ischaemic symptoms delays vascular diagnosis. Multiple cranial nerve involvement (V, VII, VIII, IX, and XII) indicates skull-base or parapharyngeal pathology adjacent to the ICA [9].

ICA dissection has been reported to cause multiple lower cranial neuropathies (IX, X, XI, and XII), Horner’s syndrome, and other stroke symptoms.

Although rare, there have been a few case reports showing the involvement of the V and VII cranial nerves [11]. There was a reported case of left ICA dissection after head trauma in 1990 with V, VII, IX, X, and XII nerve abnormalities [12].

Pulsatile tinnitus, reported in 8% of ICA dissection cases in a series of 138 patients [7], is believed to result from turbulent flow transmitted to the cochlea or mechanical compression by an expanding vessel wall.

Pulsatile tinnitus occurs when a patient perceives a rhythmic sound synchronous with their heartbeat, in vascular pathology, such as ICA dissection. It can arise through bruit transmission from turbulent blood flow, generating vibrations that are transmitted to the inner ear structure, or direct compression as an expanding dissection or pseudoaneurysm of the ICA traverses near the middle ear and inner ear structures, compressing the adjacent auditory pathways [13,14].

There have been a few case reports of ICA vascular anomaly (pseudoaneurysm or dissection) causing hearing loss [15]. The mechanism of hearing loss in ICA dissection might be more uncertain in comparison to posterior circulation territory strokes, causing deafness through impaired arterial supply of the inner ear through the cochlear artery arising from the anterior inferior cerebellar artery [16].

A putative mechanism linking ICA pathologies, such as pseudoaneurysm or dissection, to hearing loss can be either sensorineural hearing loss through local compression in its petrous segment near the cochlea and vestibular apparatus or conductive hearing loss through eustachian tube compressions as the ICA meanders through the petrous part of the temporal bone [15].

The uniqueness of our case lies in the concurrent involvement of cranial nerves V, VII, VIII, IX, and X, alongside Horner’s syndrome, due to ICA dissection.

The ICA originates from the common carotid artery and ascends through the neck without branching until it reaches the base of the skull. It enters the petrous portion of the temporal bone through the carotid canal and then courses medially toward the cavernous sinus. A periarterial sympathetic plexus surrounds the artery and lies in proximity to several cranial nerves, including the trigeminal (V), facial (VII), vestibulocochlear (VIII), glossopharyngeal (IX), vagus (X), accessory (XI), and hypoglossal (XII) nerves.

Due to this anatomical relationship, a dissection of the ICA can cause various cranial neuropathies. As the dissection propagates, it may result in localized compression, ischemia, or irritation of these adjacent structures. This accounts for the involvement of multiple cranial nerves and the presentation of symptoms such as ptosis, dysarthria, dysphagia, and facial numbness in this case.

This case underscores the importance of maintaining a high index of suspicion for vascular pathology when evaluating patients with progressive cranial neuropathies, particularly in the absence of a precise alternative diagnosis. The role of imaging was crucial in this case, with MRI/MRA ultimately confirming the ICA dissection [17]. Given the potential for devastating cerebrovascular complications, prompt recognition and initiation of antithrombotic therapy are paramount [17].

Management of ICA dissection is dictated by the presence or absence of neurologic deficits and recurrence of events [18]. Patients are often managed with either dual antiplatelet or anticoagulation. In the Cervical Artery Dissection in Stroke Study (CADISS) trial, there was no superiority in either form of medical treatment [17]. More invasive treatment options, such as stenting, were warranted if there were recurrent events or ICA occlusion [17].

Nevertheless, considering this is a solitary case report, we acknowledge the limitations in deriving broad conclusions. However, the evident temporal correlation between the patient’s symptoms, the imaging findings, and the observed improvement with treatment substantiates the probability that these events are interconnected rather than coincidental.

## Conclusions

This case highlights the diagnostic complexity of ICA dissection and the need for a multidisciplinary approach involving otolaryngology, neurology, and stroke teams. It also reinforces the importance of considering vascular causes in patients with progressive cranial nerve deficits, even in the absence of overt ischemic signs. Early imaging, particularly MRA or CTA, should be considered in patients with unexplained cranial neuropathies to avoid delays in diagnosis and treatment.
